# Harnessing emergent properties of microbial consortia for Agriculture: Assembly of the Xilonen SynCom^☆^

**DOI:** 10.1016/j.bioflm.2025.100284

**Published:** 2025-05-03

**Authors:** Gabriela Gastélum, Bruno Gómez-Gil, Gabriela Olmedo-Álvarez, Jorge Rocha

**Affiliations:** a Centro de Investigación en Alimentación y Desarrollo A.C. (CIAD) Unidad Regional Hidalgo. San Agustín Tlaxiaca, Hidalgo, 42162, Mexico; b Developmental Biology Unit and Molecular Systems Biology Unit, European Molecular Biology Laboratory, Heidelberg, Germany; c Centro de Investigación en Alimentación y Desarrollo A.C. (CIAD) Unidad Mazatlán. Acuicultura y Manejo Ambiental. Mazatlán AP711, Sinaloa, Mexico; d Departamento de Ingeniería Genética, CINVESTAV-Unidad Irapuato, Guanajuato, Mexico; e Programa de Agricultura en Zonas Áridas, Centro de Investigaciones Biológicas del Noroeste, La Paz, Baja California Sur, 23096, Mexico

**Keywords:** SynComs, Seed-endophytes, Emergent properties, Complex colony architecture, Biofilm formation, Plant-growth promoting bacteria

## Abstract

Synthetic communities (SynComs) are valuable tools for addressing microbial community assembly and function, towards their manipulation for clinical, biotechnological and agricultural applications. However, SynCom design is complicated since interactions between microbes cannot be predicted based on their individual properties. Here we aimed to assemble a functionally cohesive SynCom displaying high-order interactions, as a model to study the community-level beneficial functions of seed-endophytic bacteria from native maize landraces*,* including strains from the Bacilli class, and the *Burkholderia* and *Pseudomonas* genera. We developed a partial combinatorial, bottom-up strategy that was followed by the detection of complex colony architecture as an emergent property in co-cultures. Using this simplified approach, we tested less than 400 co-cultures from a pool of 27 strains, resulting in the assembly the *Xilonen* SynCom, which includes *Bacillus pumilus* NME155, *Burkholderia contaminans* XM7 and *Pseudomonas* sp. GW6. In this community, higher-order interactions result in complex colony architecture, which is considered a proxy of biofilm formation. Additionally, we generated protocols for absolute quantification of each member from a complex mixture. The *Xilonen* SynCom will serve as a model to study biofilm formation in community settings, and will aid in the study of the molecular and ecological basis mediating maize fertility.

## Highlights

• An accessible strategy was devised to assemble functionally cohesive SynComs.
• Maize seed endophytic bacteria were used to generate the Xilonen SynCom.
• The generated SynCom included *Bacillus*, *Pseudomonas*, and *Burkholderia* strains.
• Interactions in the Xilonen SynCom result in emergent colony biofilm architecture.
• Population dynamics within the community and pairwise interactions were evaluated.

## 1 Introduction

Microbes are essential for plant health and crop productivity, aiding the host-plant in nutrient acquisition [1], biotic and abiotic stress alleviation [2], and immunomodulation [3]. Microbial associates of plants are mainly found near or on the roots, where plant exudates sustain the growth of specific bacterial groups that contribute to these functions [[4], [5], [6], [7]]. To achieve colonization, bacterial communities organize in root-attached multi-species biofilms; interestingly, inter-specific interactions influence both biofilm formation [[8], [9], [10]] and the expression of plant-beneficial functions [[11], [12], [13], [14]].

Grasping the beneficial functions of plant-associated microbes through the development of microbial inoculants is a promising approach to enhance crop productivity while reducing the use of chemical pesticides and fertilizers [15]. Notably, inoculations with mixed microbial strains (consortia) allow increased performance in laboratory settings compared to single-strain inoculations [16]. However, scaling to field applications is a limiting step [[17], [18], [19], [20]], probably due to the insufficient incorporation of microbial ecology approaches in the development of multi-strain inoculants [21,22].

The design of microbial consortia typically involves selecting microbes based on complementary functions identified through the study of individual strains *in vitro,* and including only ‘biocompatible’ strains, *i.e.*, those that do not demonstrate antagonism when paired together [[23], [24], [25], [26], [27], [28], [29], [30], [31], [32]]. These strategies lack solid foundations since antagonism tends to be attenuated in communities with higher-order interactions [14,33]. Also, the assembly, stability, structure, and functioning of the plant microbiota are consequences of complex interactions, including metabolic cross-feeding and antagonism between community members [34]. Moreover, emergent properties of microbial communities are unpredictable from the characterization of strains in isolation [[34], [35], [36]]. Therefore, novel approaches in the design of microbial consortia for agriculture should consider the ecological processes governing community assembly and stability, as well as their impact on the resulting beneficial functions.

Synthetic communities (SynComs) have arisen as valuable experimental tools for establishing causal relationships between community members (or their genes) and community functions [37,38]. These laboratory-assembled communities are designed to represent natural communities but with reduced complexity [37], enabling precise manipulations for addressing the effect of experimental variables on community traits [39]. In this sense, generating microbial synthetic communities that enhance plant health and productivity is a strategy that circumvents many limitations in the study and application of conventional microbial consortia for agriculture [[40], [41], [42]]. However, the design of simplified, tractable (easy to manipulate), trackable (where protocols for absolute quantification of individual strains are available), and ecologically relevant SynComs remains a complex task [[34], [35], [36]]. Bottom-up approaches for SynCom design represent a rational strategy where isolates are used as building blocks [37,38], but full-combinatorial strategies are technically demanding since they depend on the preparation and analysis of a large number of co-cultures [[43], [44], [45], [46]], requiring infrastructure that is inaccessible for many non-specialized laboratories working with natural isolates.

One trait of microbial communities that could be useful for SynCom assembly is the inherent presence of emergent properties, i.e., those functions that arise from the community as a whole and are absent in individual members [34,35]. For example, some model SynComs exhibit emergent properties that may benefit host health, such as control of pathogen growth [13] and contribution to drought tolerance [12]. Similarly, co-culture studies have shown that interactions can promote the emergence of collective traits — functions that are significant in large groups of cells — such as antibiotic production [47,48], spreading motility [49], biofilm formation in liquid culture [50], or complex architecture of colony biofilms in agar [8]. These collective traits are also ecologically relevant as they can influence bacterial fitness in the rhizosphere and affect host plant health [[51], [52], [53]]. Notably, macroscopic manifestations of collective traits can often be detected visually *in vitro* [8,47,49,50]. Therefore, visual identification of a collective trait that appears as an emergent property in bacterial co-cultures could facilitate the assembly of functionally cohesive SynComs composed of members that not only coexist, but also engage in pairwise or high-order interactions that result in beneficial functions for the host plant.

To develop more successful inoculants for sustainable agriculture, recent studies have focused on bacterial communities in low-input agroecosystems, including ancestral systems such as Mesoamerican *milpas* [54,55]. Microbial associates of native maize landraces in *milpas* are essential for crop productivity since no irrigation or agrochemical inputs are used [56]. Specifically, seed-endophytic bacteria may be particularly important for the fertility of maize landraces. In this fraction of the bacteriome, Bacilli strains are diverse, abundant and capable of colonizing seedling roots [57], *Burkholderia* spp. Exhibit strong antagonistic properties, potentially influencing community assembly and biocontrol [58], and *Pseudomonas* spp. Contribute to drought tolerance in landraces from arid *milpas* [59]. Although these strains are attractive for designing multi-species inoculants, more complex experimental models are still needed to fully harness the beneficial functions of these bacteria in a community context.

Here we aimed to generate a SynCom using seed-endophytic bacteria from native maize landraces. From an initial set of 27 strains (20 strains from the Bacilli class, 4 *Pseudomonas* spp., and 3 *Burkholderia* spp.), we devised a simplified strategy for co-culture preparation and identification of emergent properties. We focused on the detection of complex colony architecture of bacterial co-cultures, a collective trait that can be identified through the visual inspection of colony morphology and is qualitatively related to the presence of an extracellular biofilm matrix (reviewed in [60]). Our screening resulted in the assembly of a three-member community named ‘*Xilonen’* after the Aztec goddess of native maize fertility [61]. Interestingly, this community is functionally cohesive since all members are needed for the emergence of complex colony architecture. The *Xilonen* community is a valuable tool for harnessing the contribution of the seed-microbiota to native maize fertility. Moreover, this work provides a framework for rapid, design-free and accessible assembly of SynComs with ecological, biotechnological, or clinical relevance.

## 2 Methods

### 2.1 Bacterial strains and culture conditions

Strains used in this study are shown in Supplementary Table S1. For all experiments, strains were streaked from cryostocks on LB agar (10 g L^−1^ tryptone, CRITERION™, 5 g L^−1^ yeast extract, Sigma-Aldrich and 5 g L^−1^ NaCl) and incubated for 2 days at 30 °C. For slow-growing strains (NME36, NME37, NME100, NME117, NME186, NME233 and NME235), one colony was picked into 50 mL of LB liquid medium and grown at 30 °C and 200 rpm for 40–42 h. For the rest of the strains, one colony was picked into 20 mL of LB liquid medium and grown at 30 °C and 200 rpm for 16–18 h. Rifampicin 50 μg/mL (rif50), tetracycline 10 μg/mL (tet10), or chloramphenicol 10 μg/mL (cam10) were added when needed.

### 2.2 Combinatorial screening of emergent colony morphology in co-cultures

**Fig. 1:**
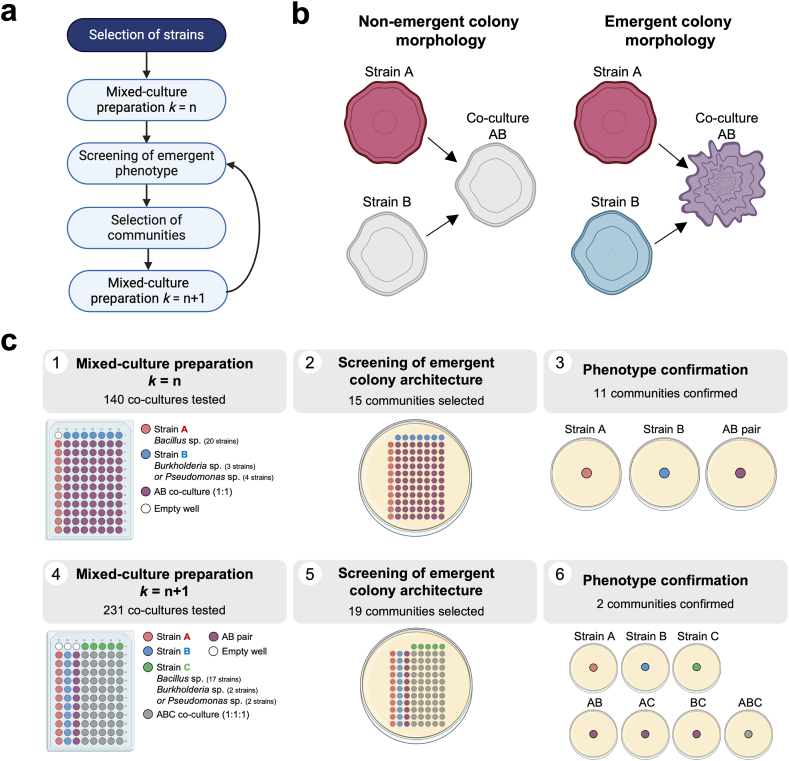
**Assembly of the *Xilonen* synthetic community through screening for emergent colony architecture resulting from interactions between seed-endophytic bacteria of native maize landraces. a,** the general strategy was based on preparation of pairwise co-cultures and screening of emergent functions for the assembly of synthetic bacterial communities of size (*k*) and subsequent addition of members for *k*+1 communities and screening. **b,** Representation of emergent colony morphology considered in our screening. Non-emergent colony morphology: morphology in the co-culture of two strains (AB) is similar to the morphology of strain A or B (left). Emergent colony morphology: morphology in the co-culture AB differs from morphologies A and B (right). **c,** Workflow followed for the assembly of the *Xilonen* synthetic community. First, AB pairs were mixed using two bacterial genera (1). After incubation, colonies were screened for emergent colony morphology (2), and phenotypes were confirmed in separate plates (3). Then, a third strain C was added to selected AB pairs (4), and co-cultures *k* = 3 were screened for emergent colony morphology (5). Finally, phenotypes of *k* = 3 communities were confirmed in separate plates against all possible single and pairwise co-cultures (6). Created with BioRender.com.

The general strategy for the bottom-up assembly of SynComs with emergent properties is shown in Fig. 1a. For the preparation of bacterial strain combinations, 10 mL of liquid cultures from all strains (Table S1) were centrifuged (4000 rpm for 10 min), washed, and suspended in 10 mL of sterile PBS. Then, the Optical Density at 600 nm (OD_600_) was adjusted to 1 ± 0.1. These suspensions were mixed into combinations in microtiter 96-well plates (AXYGEN, No. *P*-DW-11-C-S) at an equal volume ratio with a final volume of 350 μL. Once all combinations were prepared, plates were covered with a sealing mat (AXYGEN, No. AM-2 ML-RD), vortexed to ensure a homogeneous mixture, and spun briefly before re-opening. Then, 5 μL of each of the 96 wells were spotted on a previously air-dried LB plate (15 cm diameter). Inoculated spots were air-dried for 15 min, incubated at 30 °C for 3 days, and imaged using a digital camera and a copy stand. Mixed colonies were visually screened for emergent colony architecture. Emergent colony architecture was determined by comparing the morphology of a mixed colony against the morphology of all individual members (Fig. 1b). Emergent architecture of selected colonies was confirmed on separate LB plates to avoid the effect of metabolite diffusion through the agar, and communities with confirmed emergent colony morphology were used for a subsequent step of combination prep and screening (community *k* = n+1, Fig. 1a and c). All spot-inoculations were performed in triplicate.

### 2.3 Genome sequencing

DNA from *Bacillus* sp. NME155, *Burkholderia* sp. XM7, and *Pseudomonas* sp. GW6 was isolated from 2 mL of overnight liquid cultures grown in LB medium at 30 °C. Cells were harvested by centrifugation (8000 rpm, 3 min), and DNA was isolated from the pellet using the Power Soil Pro Kit (QIAGEN). The first step of the protocol was modified for our samples: pellets were suspended in 800 μL of solution CD1 and transferred to the PowerBead Pro Tube. Then, the protocol was followed as described in the manufacturer's handbook. Pair-end genome sequences (2 x 150 bp) were obtained using the Illumina Miniseq platform (Laboratorio de Genómica Microbiana, CIAD Mazatlán, Sinaloa, Mexico). Illumina reads were adapter and quality trimmed using FastQC v0.11.2. After trimming, the quality was assessed one more time using FastQC v0.11.2. Genomes were assembled using SPAdes v3.15.2 [62], and the quality of each assembly was verified using QUAST v5.0.2 and CheckM2 v.0.1.2 [63,64]. Identification of the phylogenetically closest type to each strain as well as the Average Nucleotide Identity (ANI%) for species-level identification was made using the Microbial Genomes Atlas (MiGA) web server v1.2.18.2 [65,66]. Digital DNA–DNA hybridization (dDDH) was calculated using the Type (Strain) Genome Server (TYGS) online platform [67]. Before GenBank submission, contigs <200 bp were eliminated from the genome assemblies.

### 2.4 Spontaneous rifampicin-resistant variant of *Bacillus pumilus* NME155

To recover Bp_NME155 from the *Xilonen* community, we generated a spontaneous rifampicin-resistant variant as described in Ref. [57]. Briefly, cells from 40 mL of an overnight liquid culture were harvested by centrifugation (8000 rpm and 4 °C for 10 min). The resulting pellet was suspended in 2 mL of fresh LB medium, and 500 μL of the suspension was plated on LB medium with rifampicin (50 μg/mL). Plates were air-dried and incubated in darkness at 30 °C for 2 days. Next, 5 rif^R^ colonies were isolated and a variant with a similar growth rate (LB liquid medium) and colony morphology, compared to the wild-type strain, was selected. Additionally, the presence of emergent colony architecture in the *Xilonen* community assembled with the selected rif^R^ variant was verified. The strain was grown in LB medium and preserved with 15 % glycerol at 80 °C.

### 2.5 CFU counts from colony biofilms

Colony biofilms of *Bacillus pumilus* NME155 (Bp_NME155), *Burkholderia contaminans* XM7 (Bc_XM7), and *Pseudomonas* sp. GW6 (P_GW6), were grown as described above. For inoculation of the community, cultures were mixed on a 1:1:1 vol ratio and 5 μL of the mixture was used for spot inoculation. CFU counts at day 0 were estimated by plating 10-fold serial dilutions of the liquid cultures used for spot-inoculation. For CFU counts from colony biofilms, three colonies of individual strains and the community were cut off the agar plate with a sterile scalpel and transferred to a culture tube (25 mm × 150 mm) with 5 mL of sterile PBS. Colonies were suspended by vortexing twice for 1 min, and 10-fold serial dilutions of each suspension were plated on LB, LB rif50, and LB tet10. Plates were incubated at 30 °C and CFU counts were assessed after 1–2 days. In the mixed colonies, CFUs of strain A (Bp_NME155), strain B (Bc_XM7), and strain C (P_GW6) were estimated by CFU counts on LB rif50, LB tet10, and LB, respectively.

### 2.6 Antagonistic interactions assay

Antagonistic interactions were assessed using the spot-on-lawn assay [68]. Briefly, overnight liquid cultures of strains *Bacillus pumilus* NME155 (Bp_NME155), *Burkholderia contaminans* XM7 (Bc_XM7) and *Pseudomonas* sp. GW6 (P_GW6) were washed as described above, and OD_600_ was adjusted to 1 ± 0.1. The sensitive strain was inoculated as a lawn mixed at 1:1000 vol in LB agar before pouring into Petri dishes. Plates inoculated with each “lawn-strain” were air-dried for at least 30 min, and 5 μL of each “spot-strain” were inoculated on top. Plates were air-dried for another 15 min and incubated at 30 °C. Antagonistic interactions were evaluated after 2 days of incubation and they were classified as strong antagonisms (defined halo) and weak antagonisms (dim halo). Each interaction was performed in triplicate.

### 2.7 Biofilm formation assays

Pairwise induction of biofilm formation was assessed in two separate assays, 1) in bacterial co-cultures (*k* = 2, 3) in fresh LB medium compared to single strain cultures, and 2) in single strain cultures grown in conditioned medium (2X LB medium mixed with spent medium from a different strain culture in a 1:1 ratio). For preparation of cell-free spent medium, one colony (strain A: Bp_NME155, strain B: Bc_XM7 and strain C: P_GW6) was picked into 100 mL of LB medium and grown at 30 °C and 200 rpm for 5 days. Next, cells were removed by centrifugation (10,000 rpm for 20 min, twice), and the supernatant was collected and filter-sterilized using a 0.2 μm pore size. To verify sterilization, 10 μL of each spent medium was plated on fresh LB and incubated at 30 °C for 5 days. Biofilm formation in both assays was quantified using the crystal violet method with modifications [57,69]. Briefly, overnight cultures of strains Bp_NME155, Bc_XM7, and P_GW6 were grown, washed, and adjusted to OD_600_ 1 ± 0.1. For assessing biofilm formation in bacterial co-cultures, each single strain, pairwise co-culture, and the community (mixed at an equal volume ratio) were diluted 1:100 in fresh LB medium. For the experiment using conditioned medium, each strain was diluted 1:100 in fresh LB medium or conditioned medium prepared with spent medium from each of the individual strains. Next, 100 μL of inoculated media were transferred into sterile non-tissue culture treated 96-well microtiter plates, and inoculated plates were incubated at 30 °C without agitation for 3 days. Planktonic cells were removed, plates were washed as described in Ref. [69], and biofilms were stained with 250 μL of 0.1 % (w/v) crystal violet for 10 min at room temperature. After incubation, plates were washed twice and stained biofilms were dissolved with 250 μL of 30 % (v/v) glacial acetic acid for 15 min. For quantification, 125 μL of the dissolved and stained biofilm was transferred to a clear flat-bottom microtiter plate, and OD_600_ was measured using a plate reader (Varioskan Lux, Thermo Scientific). For both assays, representative microtiter wells were imaged using an Axio Zoom.V16 stereo microscope with an incorporated Axiocam 105 color 429 (Carl Zeiss Microscopy, Oberkochen, Germany) before quantifying biofilm formation. Wells with non-inoculated medium were used as control.

## 3 Results

### 3.1 Assembly of the *Xilonen* synthetic community

To generate a synthetic community (SynCom), we established a bottom-up combinatorial strategy based on the detection of emergent properties in bacterial co-cultures, focusing on the visual detection of collective traits. Specifically, we sought to assemble a SynCom with emergent complex colony architecture, a type of colony morphology characterized by the presence of elevated wrinkles or bundles throughout the colony [60,70,71]. Importantly, complex colony architecture results from the production of extracellular matrix components such as polysaccharides and proteins [[72], [73], [74], [75], [76]] and its widely considered as a proxy for biofilm formation [71,[77], [78], [79], [80]].

Using 27 seed-endophytic bacterial strains from maize landraces from our collection: 20 strains from the Bacilli class, 3 *Burkholderia* sp., and 4 *Pseudomonas* sp. (Supplementary Table S1), we prepared co-cultures for screening emergent colony architecture. It is important to note that a full combinatorial bottom-up approach testing all combinations of size *k* = {2, 3, 4} from 27 strains would entail preparing and screening 20,826 co-cultures. In our approach, we used a partial-combinatorial screening, reducing the number of co-cultures by 1) initially testing co-cultures of *k =* 2 with two different bacterial genera (one Bacilli and one *Burkholderia* spp. or *Pseudomonas* spp.) and 2) selecting specific co-cultures of *k* = 2 with emergent colony morphology (including but not limited to complex colony architecture), before the introduction of an additional strain to assemble communities of *k* = 3, and repeating the process (Fig. 1a). Colony morphology encompasses characteristics like size, shape, elevation, edge appearance, color, optical properties, and texture; we considered as emergent any morphology in a co-culture colony that differed from that of the individual members (Fig. 1b) or subgroups.

**Fig. 2:**
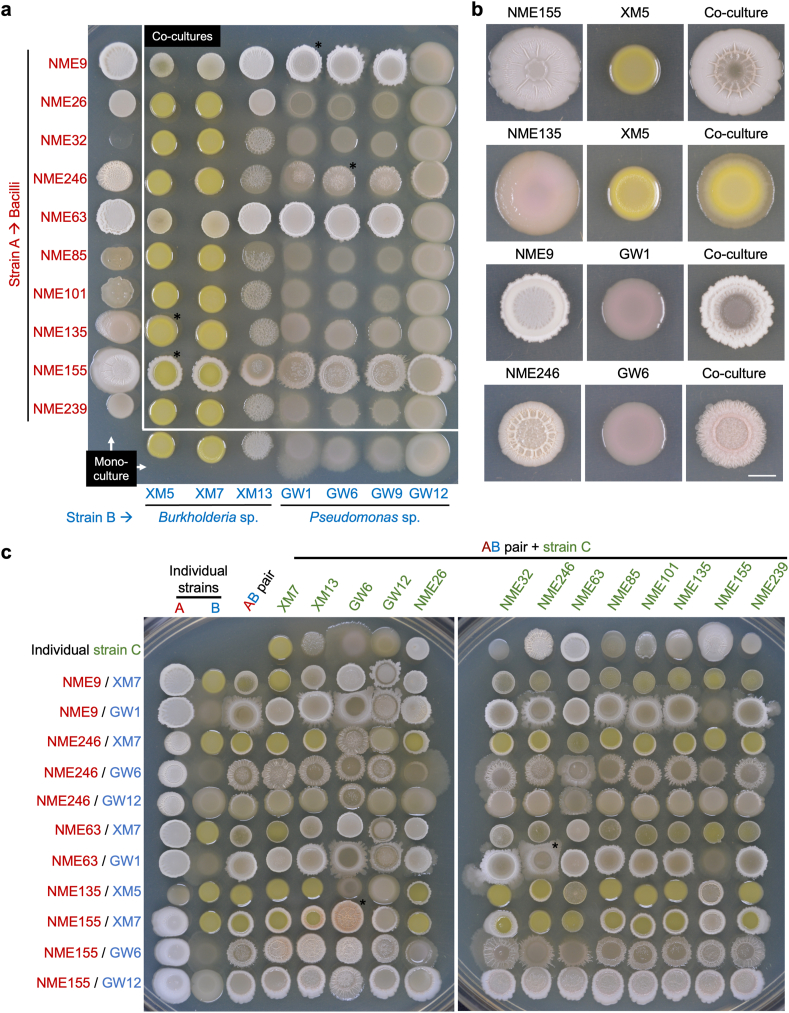
**Screening of communities *k* = 2, 3. a,** Representative plate for screening 70 co-cultures of *k* = 2 after 3 days of incubation. Colonies with asterisks are shown in part b. **b,** Representative communities of *k* = 2 with emergent colony morphology, selected for a subsequent step of combination prep and screening. Colonies were grown in separate LB plates and pictures were taken after 3 days of incubation at 30 °C. Scale bar: 5 mm. **c,** Representative plates for screening 143 co-cultures of *k* = 3 after 3 days of incubation. The phenotype of colonies with asterisks was confirmed in separate plates.

First, we prepared co-cultures of *k* = 2 including one of 20 Bacilli (strain A) and one of seven *Burkholderia* sp. or *Pseudomonas* sp. (strain B) (Panel 1 of Fig. 1c, Fig. 2a). From the 140 co-cultures, we detected 15 AB pairs exhibiting emergent colony morphology (Panel 2 of Fig. 1c). When these co-cultures were tested in separate agar plates, 11 communities with emergent morphology were confirmed (Panel 3 of Fig. 1c). Representative communities of *k* = 2 with emergent colony morphology are shown in Fig. 2b, including co-cultures with modified architecture (e.g., NME155 + XM5 and NME246 + GW6), distinct shape and color (e.g., NME135 + XM5), and perceived spatial segregation (e.g., NME9 + GW1) (Fig. 2b).

**Fig. 3:**
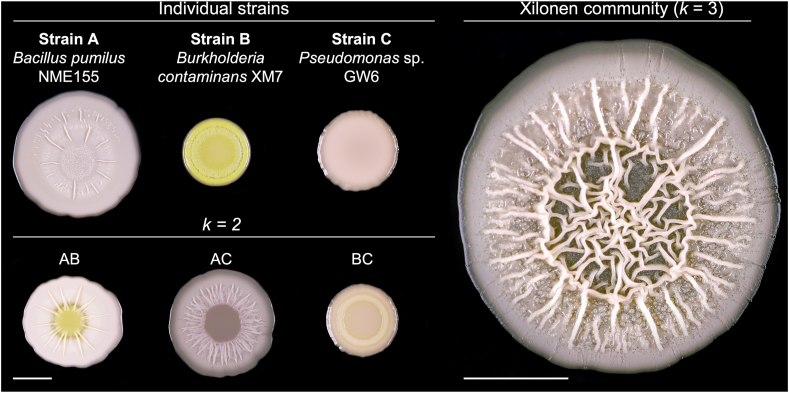
***Xilonen* synthetic community. Complex colony architecture emerges only through the interaction of the three strains in the community.** Colonies of individual strains that form the synthetic community, colonies of pairwise co-cultures (*k* = 2), and the *Xilonen* community (*k* = 3). Each colony was grown individually in separate LB plates. Pictures were taken after 3 days of incubation at 30 °C. Both scale bars are 5 mm.

Next, we screened 231 co-cultures of *k* = 3 that resulted from adding 21 strains (strain C) to 11 confirmed AB communities (Panel 4 of Fig. 1c, Fig. 2c). In this step, we identified 19 ABC co-cultures with emergent colony morphology (Panel 5 of Fig. 1c). When confirming the emergent phenotype in separate plates, we found that only two ABC communities displayed emergent colony morphology. The colony morphology of both ABC co-cultures differed from their respective individual strains (A, B, C) and pairwise co-cultures (AB, AC, and BC, Panel 6 of Fig. 1c); therefore, they were considered as emergent properties resulting from higher-order interactions. One community displayed emergent spreading motility; it consisted of *Bacillus* sp. NME63, *Pseudomonas* sp. GW1, and *Bacillus* sp. NME246 (Supplementary Fig. S1). The other community exhibited emergent complex colony architecture (Fig. 3). This community included the strains *Bacillus* sp. NME155, *Burkholderia* sp. XM7 and *Pseudomonas* sp. GW6 (Fig. 3). We also screened 24 co-cultures of *k* = 4 by adding 12 strains to both communities of *k* = 3, but we did not observe an emergent colony morphology in this step (not shown).

**Fig. 4:**
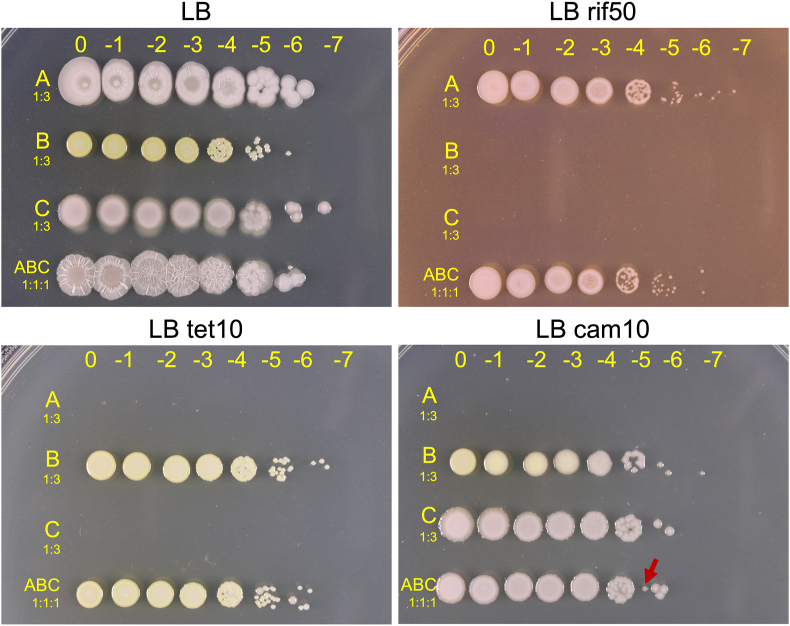
**Selective media for individual CFU counts of each member of the community.** Spot inoculations of 10-fold serial dilutions of liquid cultures of individual strains and mixed cultures. Overnight bacterial liquid cultures were washed with PBS and the optical density at 600 nm was adjusted to 1 ± 0.1. Washed bacterial cultures were mixed at a 1:1:1 vol ratio, while individual strains were diluted 1:3 in sterile PBS. Five μL of 10-fold serial dilutions were spotted on LB and each selective medium. Rifampicin 50 μg/mL (rif50); tetracycline 10 μg/mL (tet10) or chloramphenicol 10 μg/mL (cam10) were added to LB medium to recover strains A (Bp_NME155), B (Bc_XM13), and C (P_GW6) respectively. The arrow in the LB cam10 plate indicates small colonies corresponding to strain B, which are not considered for CFU counts in this selective medium.

**Fig. 5:**
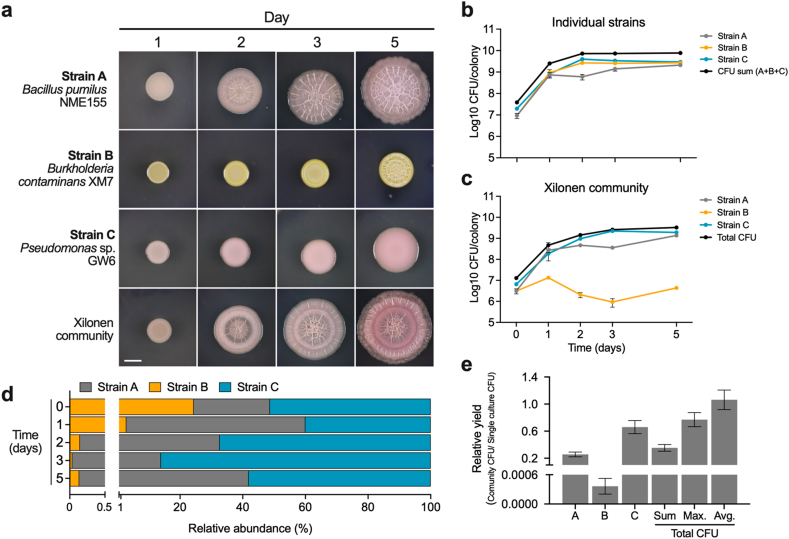
**Growth dynamics of individual strains in the *Xilonen* SynCom. a,** Colony development of individual strains and the mixed community. Scale bar, 5 mm **b,** CFU counts from colonies of individual strains. **c,** CFU counts of individual strains in the *Xilonen* community. **d,** Relative abundance of each member of the community on days 0, 1, 2, 3, and 5. **e,** Relative yield of individual strains and total cells in the *Xilonen* community, compared to individually grown strains on day 3. Total CFUs in (e) are shown relative to the sum of individual strains (Sum), to the strain with maximum individual yield (Max.) and the average yield per colony (Avg.). The average of three replicates ± SD is shown.

**Fig. 6:**
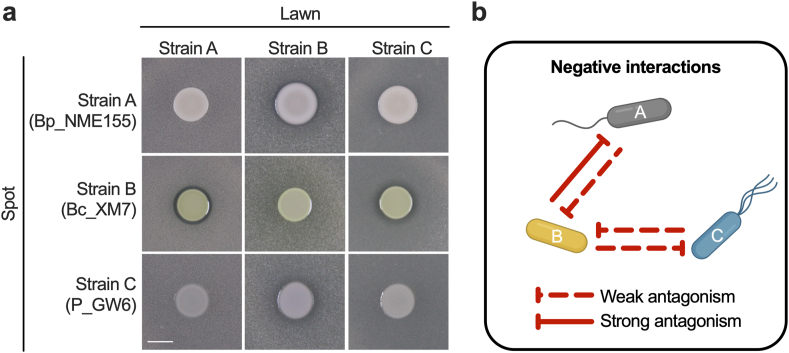
**Pairwise negative (antagonism) interactions between strains in the *Xilonen* SynCom. a,** Antagonistic interactions using the spot-on-lawn assay. Pictures were taken 24 h after incubation at 30 °C. Scale: 5 mm **b,** Schematic representation of pairwise antagonistic interactions. Strain A: *Bacillus pumilus* NME155 (Bp_NME155); Strain B: *Burkholderia contaminans* XM7 (Bc_XM7); Strain C: *Pseudomonas* sp. GW6 (P_GW6).

In summary, our screening encompassed only 395 co-cultures, resulting in the identification of one community of *k* = 3 that exhibited an emergent complex colony architecture and included the three bacterial taxa considered (Fig. 3). We named this community *Xilonen*, and it was further characterized.

### 3.2 Genomic sequence and species-level identification of the members of the *Xilonen* community

The *Xilonen* synthetic community includes *Bacillus* sp. NME155, *Burkholderia* sp. XM7, and *Pseudomonas* sp. GW6 strains, which were preliminarily identified at the genus level in previous studies [[57], [58], [59]]. For species-level identification, we carried out genomic DNA sequencing using Illumina Miniseq and *de novo* assembly. Sequencing, assembly, and annotation statistics are summarized in Supplementary Table S1. From the genomic data, two of the strains were identified at the species level as *Bacillus pumilus* (Bp_NME155) and *Burkholderia contaminans* (Bc_XM7). Their Average Nucleotide Identity (ANI%) and digital DNA-DNA Hybridization (dDDH%) values with their phylogenetically closest type strain were 95.36 % and 63.9 % for Bp_NME155 (*B. pumilus* NCTC10337; NCBI RefSeq assembly no. GCF_900186955.1); and 99.58 % and 98.9 % for Bc_XM7 (*B. contaminans* LMG 23361; NCBI RefSeq assembly no. GCF_000987075.1), respectively (Table S1). The *Pseudomonas* sp. GW6 strain could only be identified at the genus level since it had an ANI of only 91.89 % and 44.8 % dDDH when compared with its closest relative *Pseudomonas sediminis* PI11 (NCBI RefSeq assembly no. GCF_002741105.1) (Supplementary Table S2). These values are below the threshold recommended for species identification (≥95 % ANI and ≥70 % dDDH) [81,82].

Our sequencing efforts allowed the assembly and annotation of strains Bp_NME155 and P_GW6 (GenBank Whole Genome Sequence Acc. No. JBKAED000000000 and JBLUOL000000000, respectively). Results from the quality evaluation of the assemblies showed that Bp_NME155 and P_GW6 had 100 % completeness with 0.03 % and 0.38 % contamination, respectively (Supplementary Table S2). For Bp_NME155 4056 coding sequences, 86 RNAs, and 1385 annotated genes were predicted; P_GW6 exhibited 5489 coding sequences, 79 RNAs, and 1591 annotated genes (Supplementary Table S2). For Bc_XM7, sequencing allowed its identification at the species level; however, assembly quality was insufficient (N50 value of 1,117, completeness of 66.39 % and contamination of 7.48 %; Supplementary Table S2) and require further sequencing efforts [83,84].

### 3.3 Developing a protocol for absolute quantification of the *Xilonen* SynCom members from the mixed colony biofilm

To examine the growth dynamics in the community, we devised a strategy to recover and quantify each strain from the mixed colony biofilm. Differential antibiotic resistance was deemed a useful approach. First, we performed Minimum Inhibitory Concentration (MIC) assays using eight antibiotics (Supplementary Fig. S2), and results indicated that tetracycline and chloramphenicol could be used to generate selective media for Bc_XM7 and P_GW6, respectively. Next, we tested if we could quantify these two strains from a mixture of independently grown liquid cultures. The addition of 10 μg/ml of tetracycline allowed the selection of Bc_XM7, completely inhibiting the growth of Bp_NME155 and P_GW6 (Fig. 4). Also, 10 μg/ml of chloramphenicol was useful to quantify P_GW6 by inhibiting the growth of Bp_NME155 and decreasing the size of Bc_XM7 colonies. In both cases, the corresponding strain grew to similar CFUs compared to medium without antibiotics (Fig. 4).

Our MIC assays did not allow the generation of a selective medium for Bp_NME155. Hence, for this strain, we generated a rifampicin-resistant variant (rif^R^) to enable its selection and quantification from the colony biofilm (see methods). We verified that this variant maintained the colony morphology of the WT strain, and allowed the development of emergent complex colony architecture when incorporated in the *Xilonen* community (Supplementary Fig. S3). The addition of 50 μg/mL of rifampicin allowed the quantification of Bp_NME155 rif^R^ from the mixture of three strains (Fig. 4).

Next, we tested whether the selective media allowed accurate CFU counts in suspensions obtained from colony biofilms of individual strains. Strains Bp_NME155 and Bc_XM7 showed consistent CFU counts in media with and without the corresponding antibiotics when measured from colony biofilms (not shown). However, the addition of chloramphenicol resulted in a decrease of CFU counts of P_GW6 compared to media without antibiotics (Supplementary Fig. S4) when it was quantified from colony biofilms. This phenomenon likely affects the quantification of P_GW6 from the *Xilonen* community; however, our preliminary assays showed that P_GW6 was the dominant species in the mixed biofilm, and it was possible to quantify its CFUs in non-selective medium from its distinct colony morphology.

### 3.4 Growth dynamics in the *Xilonen* SynCom

We used the devised strategy to evaluate the growth dynamics of the three strains during the development of the mixed colony biofilm by following CFUs for 5 days, compared to growth in single colonies (Fig. 5a). In the individual inoculations, colonies of Bc_XM7 (strain B) and P_GW6 (strain C) presented a maximum of 2.66 × 10^9^, and 3.96 × 10^9^ CFU/colony on day 2, respectively; Bp_NME155 (strain A) presented a maximum of 2.1 × 10^9^ CFU/colony on day 5 (Fig. 5b). Interactions in the community context caused changes in the growth dynamics of the three strains (Fig. 5b and c). The maximum total CFU count was observed on day five with a yield of 3.39 × 10^9^ CFU/colony (1.36 × 10^9^ CFU of strain A, 4.36 × 10^6^ CFU of strain B and 1.9 × 10^9^ CFU of strain C) (Fig. 5c). Strain C dominated the community during most days of biofilm development ranging from 40 % to 86.08 %. Abundance of strain A ranged from 13.87 % to 57.14 %, dominating the community on day two, while strain B was the least abundant in the community representing only between 0.036 % and 2.85 % (Fig. 5d).

The characteristic emergent complex colony architecture of the *Xilonen* SynCom was observed on day 3 (Fig. 5a); however, interactions in the community and the emergence of colony architecture were not accompanied by growth induction [85] of the community or the individual strains. On day 3, the growth of strains A, B, and C in the community represented only 26 %, 0.036 %, and 66.33 %, respectively, compared to their growth as individual colonies (Fig. 5e). The total yield in the *Xilonen* community on day 3 (2.59 × 10^9^ CFU/colony) was only 35 % of the expected yield from the sum of the three strains grown individually, 77 % of that of the strain with greater individual yield (strain C), and 106 % of the average yield of individual strains (Fig. 5e).

### 3.5 Pairwise antagonistic interactions

Emergent properties in microbial communities result from higher-order interactions and environmental conditions, all of which drive community structure, function, assembly, stability, and evolution. However, the analysis of lower-order (pairwise) interactions can be useful to explain these complex processes [35,[86], [87], [88]]. To gain insights into the interactions driving the emergent properties of the *Xilonen* SynCom, we evaluated pairwise negative interactions through an antagonism assay using the spot-on-lawn method (Fig. 6a). From the six possible pairwise combinations we found four antagonistic interactions. Strains A and B antagonized each other through weak and strong interactions, respectively; while strains B and C antagonized mutually through weak interactions (Fig. 6a and b).

### 3.6 Pairwise induction of biofilm formation

**Fig. 7:**
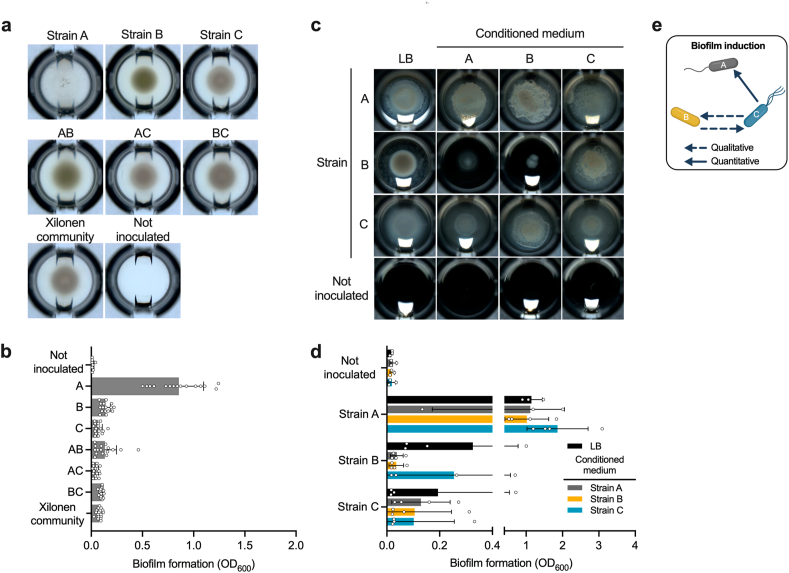
**Pairwise induction of biofilm formation in the community in standing liquid culture. a,** Microtiter wells showing the growth of single strains, pairwise co-cultures, and the three-member SynCom in standing liquid LB medium. Plates were imaged after 3 days of incubation at 30 °C. **b,** Biofilm formation of cultures shown in (a) was quantified using the crystal violet method; average, standard deviation, and individual values for 16 replicates are shown. **c,** Microtiter wells showing the development of pellicles of each strain grown individually in fresh LB medium and in conditioned medium (see methods). Plates were imaged after 3 days of incubation at 30 °C. **d,** Biofilm formation of cultures shown in (c) was quantified using the crystal violet method; average, standard deviation, and individual values for 3–4 replicates are shown. **e,** Schematic representation of pairwise induction of biofilm formation. Strain A: *Bacillus pumilus* NME155; Strain B: *Burkholderia contaminans* XM7 and Strain C: *Pseudomonas* sp. GW6. Dots represent individual replicates. (For interpretation of the references to color in this figure legend, the reader is referred to the Web version of this article.)

Complex colony architecture is mediated by molecules that also participate in biofilm formation [72,89]. For this reason, we evaluated pairwise induction of biofilm formation in standing liquid culture as a more direct approach to address this emergent property of the *Xilonen* SynCom. Biofilm formation was determined qualitatively by visually detecting pellicle biofilms in the liquid-air interface, and quantitatively using the crystal violet assay which targets surface-adhered biofilms (adhesion to microplate wells) [69]. First, we compared the biofilm formation of individual strains, pairwise co-cultures, and the three-member community. Notably, only strain A formed a pellicle in single strain culture (Fig. 7a). When biofilm surface-adhesion was quantified, individual strains A, B, and C, exhibited 0.9, 0.13, and 0.06 mean OD_600_, respectively (Fig. 7b). Interestingly, we found that biofilm adhesion was not increased in pairwise co-cultures (mean OD_600_ of 0.13, 0.04, and 0.09 for AB, AC, and BC, respectively). The three-strain ABC co-culture also failed to form a robust pellicle biofilm in standing liquid culture (mean OD_600_ = 0.07) (Fig. 7b). These findings suggest that the synthesis of compounds related to biofilm formation in the *Xilonen* community co-culture is contingent upon the structure provided by the agar medium.

We also evaluated pairwise induction of biofilm formation by cultivating single strains in conditioned medium (fresh LB amended with cell-free spent medium at a 1:1 ratio), compared to fresh LB alone; this approach tested if metabolites secreted by one strain could induce biofilm formation by cells of a different strain. Strain A developed a pellicle across all conditions (Fig. 7c). For strain B, a pellicle was developed in the presence of conditioned medium from strain C (Fig. 7c and e), and bacterial growth was decreased in conditioned medium from strain A and B (self), compared to fresh LB (Fig. 7c). Strain C developed a pellicle in conditioned medium of strain B (Fig. 7c and e). When biofilm surface-adhesion was quantified, strain A presented high biofilm adhesion in all conditions (mean OD_600_ from 1.01 to 1.86), showing a slight increase in the presence of conditioned medium from strain C compared to fresh LB (mean OD_600_ of 1.86 and 1.14, respectively) (Fig. 7d and e). For strains B and C, biofilm adhesion was not induced by any conditioned medium (Fig. 7d). This assay shed light into pairwise interactions (Fig. 7e) driven by secreted cues that could mediate emergent colony architecture in the *Xilonen* SynCom during development in semi-solid LB medium.

## 4 Discussion

We present the assembly of a three-member synthetic community (SynCom) with emergent complex colony architecture. This SynCom was named *Xilonen* after the Aztec goddess of young maize and fertility, since it is composed of three strains belonging to ecologically relevant taxa within the seed-endophytic bacteriome of Mexican maize landraces [[57], [58], [59]]. Understanding the contribution of the microbiota to plant health and the subsequent development of beneficial inoculants requires novel approaches that consider microbial interactions within microbial communities and with their hosts [34,35]. Our strategy resulted in the generation of a functionally cohesive SynCom [90,91], where all members are needed for the expression of the emergent traits in the community. The *Xilonen* community could serve as model to understand the molecular and ecological basis for beneficial plant-microbe interactions of bacterial guilds [92] towards the generation of more effective bioinoculants for agriculture. Additionally, this rapid, design-free strategy for SynCom assembly offers a versatile approach for gaining a mechanistic understanding of the main drivers of microbial interactions and emergent functions in microbial communities, as well as for obtaining consortia with diverse applications.

Efforts to develop microbial-based products for sustainable agriculture have incorporated the design of microbial consortia to enhance the effects of individual plant growth-promoting bacteria (PGPB) [21,22]. Yet, these formulations generally overlook ecological traits such as microbial interactions and their influence on community functions [16,21,22]. For instance, a common assumption is that combining ‘biocompatible’ PGPB strains (i.e., strains lacking antagonistic interactions), or strains with complementary plant-beneficial functions will result in additive or synergistic traits [[23], [24], [25], [26], [27], [28], [29], [30], [31], [32]]. However, community functions cannot be predicted by the sum of its parts [93]. Furthermore, negative interactions are common during assembly and contribute to community stability [86,94]. Here we show that strains in the *Xilonen* community display pairwise negative interactions, which could contribute to the coexistence and dynamics of the three strains in a community setting. Noteworthy, if we had initially focused on avoiding negative interactions between strains to select isolates for our screening, we would not have generated the *Xilonen* community. Hence, implementing synthetic ecology approaches (e.g., design-free, bottom-up approaches) in the development of microbial inoculants, is a promising approach to obtain ecologically-relevant and functionally robust microbial products that thrive in varying environmental conditions and in the presence of native soil microbiota. Future studies will address the significance of emergent biofilm formation in the *Xilonen* SynCom in more ecologically relevant setting such as maize roots. Altogether, we expect that these insights will contribute to more successful incorporation of bacterial communities in sustainable agriculture.

Colony architecture in the *Xilonen* community depends on the coexistence of its three members. *In vitro* colony architecture is considered a proxy for biofilm formation in model bacterial species [73,80,95] since these macroscopic structures are associated to the presence of molecules that constitute the extracellular matrix in which cells are embedded – such as exopolysaccharides (EPS), extracellular proteins, and surfactants – which are also found in biofilms in nature [96]. A structured biofilm generates heterogeneous microenvironments where physical and biological factors influence strain fitness and shape the community function, including its macroscopic properties [97,98]. Interestingly, colony architecture in the *Xilonen* community was accompanied by decreased growth of individual strains compared to single inoculations, particularly Bc_XM7. This suggest that the emergent complexity of colony architecture could be related to a trade-off between increased production of biofilm components and decreased growth. However, the molecular mechanisms underlying this emergent morphology, and the effects on fitness of individual strains upon different environmental stressors is yet to be addressed in future experiments. Additionally, further research on functional dynamics of the *Xilonen* SynCom is crucial for understanding the properties of mixed biofilms composed by these non-model bacteria.

## 5 Conclusion

Our study introduces a novel three-strain synthetic community (SynCom) named “*Xilonen*”. By tapping into the richness of seed-endophytic bacteria from maize landraces, we constructed SynComs that mirror complex functions observed in natural communities. Our systematic bottom-up approach prioritizes the selection of strains based on anticipated properties of the assembled community, which could translate to enhanced performance in agricultural settings. Notably, our focus on biofilm formation, a key factor in root colonization, underscores the significance of these SynComs in agricultural applications. The *Xilonen* community exemplifies the potential of combinatorial screenings to identify communities with emergent properties aligned with desired outcomes. These SynComs represent invaluable model systems for advancing our understanding of how microbial communities influence maize development, paving the way for innovative agricultural practices and sustainable crop management strategies.

## CRediT authorship contribution statement

**Gabriela Gastélum:** Writing – review & editing, Writing – original draft, Visualization, Software, Methodology, Investigation, Formal analysis, Data curation, Conceptualization. **Bruno Gómez-Gil:** Writing – review & editing, Validation, Supervision, Software, Resources, Investigation, Funding acquisition, Data curation. **Gabriela Olmedo-Álvarez:** Writing – review & editing, Validation, Supervision, Resources, Funding acquisition, Conceptualization. **Jorge Rocha:** Writing – review & editing, Writing – original draft, Visualization, Validation, Supervision, Resources, Project administration, Funding acquisition, Conceptualization.

## Declaration of competing interest

The authors declare that they have no known competing financial interests or personal relationships that could have appeared to influence the work reported in this paper.

## Data Availability

Data will be made available on request.

## References

[1] Richardson A.E., Simpson R.J. (2011). Soil microorganisms mediating phosphorus availability update on microbial phosphorus. Plant Physiol.

[2] Shameer S., Prasad T. (2018). Plant growth promoting rhizobacteria for sustainable agricultural practices with special reference to biotic and abiotic stresses. Plant Growth Regul.

[3] Pieterse C.M.J., Zamioudis C., Berendsen R.L., Weller D.M., Van Wees S.C.M., Bakker P.A.H.M. (2014). Induced systemic resistance by beneficial microbes. Annu Rev Phytopathol.

[4] Haichar F. el Z., Marol C., Berge O., Rangel-Castro J.I., Prosser J.I., Balesdent J. (2008). Plant host habitat and root exudates shape soil bacterial community structure. ISME J.

[5] Tiziani R., Miras-Moreno B., Malacrinò A., Vescio R., Lucini L., Mimmo T. (2022). Drought, heat, and their combination impact the root exudation patterns and rhizosphere microbiome in maize roots. Environ Exp Bot.

[6] Blagodatskaya E., Blagodatsky S., Anderson T.-H., Kuzyakov Y. (2014). Microbial growth and carbon use efficiency in the rhizosphere and root-free soil. PLoS One.

[7] Ling N., Wang T., Kuzyakov Y. (2022). Rhizosphere bacteriome structure and functions. Nat Commun.

[8] Yannarell S.M., Grandchamp G.M., Chen S.-Y., Daniels K.E., Shank E.A. (2019). A dual-species biofilm with emergent mechanical and protective properties. J Bacteriol.

[9] Burmølle M., Webb J.S., Rao D., Hansen L.H., Sørensen S.J., Kjelleberg S. (2006). Enhanced biofilm formation and increased resistance to antimicrobial agents and bacterial invasion are caused by synergistic interactions in multispecies biofilms. Appl Environ Microbiol.

[10] Lee K.W.K., Periasamy S., Mukherjee M., Xie C., Kjelleberg S., Rice S.A. (2014). Biofilm development and enhanced stress resistance of a model, mixed-species community biofilm. ISME J.

[11] Hassani M.A., Durán P., Hacquard S. (2018). Microbial interactions within the plant holobiont. Microbiome.

[12] Yang N., Nesme J., Røder H.L., Li X., Zuo Z., Petersen M. (2021). Emergent bacterial community properties induce enhanced drought tolerance in Arabidopsis. NPJ Biofilms Microbiomes.

[13] Niu B., Paulson J.N., Zheng X., Kolter R. (2017). Simplified and representative bacterial community of maize roots. Proc Natl Acad Sci U S A.

[14] Lozano G.L., Bravo J.I., Garavito Diago M.F., Park H.B., Hurley A., Peterson S.B. (2019). Introducing THOR, a model microbiome for genetic dissection of community behavior. mBio.

[15] Naik K., Mishra S., Srichandan H., Singh P.K., Sarangi P.K. (2019). Plant growth promoting microbes: potential link to sustainable agriculture and environment. Biocatal Agric Biotechnol.

[16] Liu X., Mei S., Salles J.F. (2023). Inoculated microbial consortia perform better than single strains in living soil: a meta-analysis. Appl Soil Ecol.

[17] Finkel O.M., Castrillo G., Paredes S.H., González I.S., Dangl J.L. (2017). Understanding and exploiting plant beneficial microbes. Curr Opin Plant Biol.

[18] Bacilio M., Moreno M., Lopez-Aguilar D.R., Bashan Y. (2017). Scaling from the growth chamber to the greenhouse to the field: demonstration of diminishing effects of mitigation of salinity in peppers inoculated with plant growth-promoting bacterium and humic acids. Appl Soil Ecol.

[19] Bashan Y., de-Bashan L.E., Prabhu S.R., Hernandez J.-P. (2014). Advances in plant growth-promoting bacterial inoculant technology: formulations and practical perspectives (1998–2013). Plant Soil.

[20] Sergaki C., Lagunas B., Lidbury I., Gifford M.L., Schäfer P. (2018). Challenges and approaches in microbiome research: from fundamental to applied. Front Plant Sci.

[21] Kaminsky L.M., Trexler R.V., Malik R.J., Hockett K.L., Bell T.H. (2019). The inherent conflicts in developing soil microbial inoculants. Trends Biotechnol.

[22] Rilling J.I., Acuña J.J., Nannipieri P., Cassan F., Maruyama F., Jorquera M.A. (2019). Current opinion and perspectives on the methods for tracking and monitoring plant growth‒promoting bacteria. Soil Biol Biochem.

[23] Devi R., Kaur T., Kour D., Yadav A.N. (2022). Microbial consortium of mineral solubilizing and nitrogen fixing bacteria for plant growth promotion of amaranth (Amaranthus hypochondrius L.). Biocatal Agric Biotechnol.

[24] Kaur T., Devi R., Kumar S., Sheikh I., Kour D., Yadav A.N. (2022). Microbial consortium with nitrogen fixing and mineral solubilizing attributes for growth of barley (Hordeum vulgare L.). Heliyon.

[25] Kaur T., Devi R., Kumar S., Kour D., Yadav A.N. (2023). Plant growth promotion of pearl millet (Pennisetum glaucum L.) by novel bacterial consortium with multifunctional attributes. Biologia.

[26] Kumar A., Maurya B.R., Raghuwanshi R. (2021). The microbial consortium of indigenous rhizobacteria improving plant health, yield and nutrient content in wheat (Triticum aestivum). J Plant Nutr.

[27] la Vega-Camarillo D., Sotelo-Aguilar J., Rios-Galicia B., Mercado-Flores Y., Arteaga-Garibay R., Villa-Tanaca L. (2023). Promotion of the growth and yield of Zea mays by synthetic microbial communities from Jala maize. Front Microbiol.

[28] Magallon-Servin P., Antoun H., Taktek S., de-Bashan L.E. (2020). Designing a multi-species inoculant of phosphate rock-solubilizing bacteria compatible with arbuscular mycorrhizae for plant growth promotion in low-P soil amended with PR. Biol Fertil Soils.

[29] Molina-Romero D., Baez A., Quintero-Hernández V., Castañeda-Lucio M., Fuentes-Ramírez L.E., Bustillos-Cristales M. del R. (2017). Compatible bacterial mixture, tolerant to desiccation, improves maize plant growth. PLoS One.

[30] Negi R., Kaur T., Devi R., Kour D., Yadav A.N. (2022). Assessment of nitrogen-fixing endophytic and mineral solubilizing rhizospheric bacteria as multifunctional microbial consortium for growth promotion of wheat and wild wheat relative Aegilops kotschyi. Heliyon.

[31] Paganin P., Isca C., Tasso F., Calandrelli T., Migliore G., Marras P.A. (2023). A bacterial formula with native strains as alternative to chemical fertiliser for tomato crop. Plant Growth Regul.

[32] Pandey P., Maheshwari D.K. (2007). Two-species microbial consortium for growth promotion of Cajanus cajan. Curr Sci.

[33] Aguilar-Salinas B., Olmedo-Álvarez G. (2023). A three-species synthetic community model whose rapid response to antagonism allows the study of higher-order dynamics and emergent properties in minutes. Front Microbiol.

[34] Konopka A. (2009). What is microbial community ecology?. ISME J.

[35] Madsen J.S., Sørensen S.J., Burmølle M. (2018). Bacterial social interactions and the emergence of community-intrinsic properties. Curr Opin Microbiol.

[36] van den Berg N.I., Machado D., Santos S., Rocha I., Chacón J., Harcombe W. (2022). Ecological modelling approaches for predicting emergent properties in microbial communities. Nat Ecol Evol.

[37] Großkopf T., Soyer O.S. (2014). Synthetic microbial communities. Curr Opin Microbiol.

[38] Vorholt J.A., Vogel C., Carlström C.I., Müller D.B. (2017). Establishing causality: opportunities of synthetic communities for plant microbiome research. Cell Host Microbe.

[39] Mee M.T., Collins J.J., Church G.M., Wang H.H. (2014). Syntrophic exchange in synthetic microbial communities. Proc Natl Acad Sci.

[40] Martins S.J., Pasche J., Silva H.A.O., Selten G., Savastano N., Abreu L.M. (2023). The use of synthetic microbial communities to improve plant health. Phytopathology.

[41] Pradhan S., Tyagi R., Sharma S. (2022). Combating biotic stresses in plants by synthetic microbial communities: principles, applications and challenges. J Appl Microbiol.

[42] Trivedi P., Leach J.E., Tringe S.G., Sa T., Singh B.K. (2020). Plant–microbiome interactions: from community assembly to plant health. Nat Rev Microbiol.

[43] Kehe J., Kulesa A., Ortiz A., Ackerman C.M., Thakku S.G., Sellers D. (2019). Massively parallel screening of synthetic microbial communities. Proc Natl Acad Sci U S A.

[44] Nai C., Meyer V. (2018). From axenic to mixed cultures: technological advances accelerating a paradigm shift in microbiology. Trends Microbiol.

[45] Colarusso A.V., Goodchild-Michelman I., Rayle M., Zomorrodi A.R. (2021). Computational modeling of metabolism in microbial communities on a genome-scale. Curr Opin Syst Biol.

[46] Emmenegger B., Massoni J., Pestalozzi C.M., Bortfeld-Miller M., Maier B.A., Vorholt J.A. (2023). Identifying microbiota community patterns important for plant protection using synthetic communities and machine learning. Nat Commun.

[47] McClung D.J., Du Y., Antonich D.J., Bonet B., Zhang W., Traxler M.F. (2022). Harnessing rare actinomycete interactions and intrinsic antimicrobial resistance enables discovery of an unusual metabolic inhibitor. mBio.

[48] Pishchany G., Mevers E., Ndousse-Fetter S., Horvath Jr DJ., Paludo C.R., Silva-Junior E.A. (2018). Amycomicin is a potent and specific antibiotic discovered with a targeted interaction screen. Proc Natl Acad Sci.

[49] McCully L.M., Bitzer A.S., Seaton S.C., Smith L.M., Silby M.W. (2019). Interspecies social spreading: interaction between two sessile soil bacteria leads to emergence of surface motility. mSphere.

[50] Ren D., Madsen J.S., Sørensen S.J., Burmølle M. (2015). High prevalence of biofilm synergy among bacterial soil isolates in cocultures indicates bacterial interspecific cooperation. ISME J.

[51] Van Acker H., Van Dijck P., Coenye T. (2014). Molecular mechanisms of antimicrobial tolerance and resistance in bacterial and fungal biofilms. Trends Microbiol.

[52] Zhang M., Pereira e Silva M. de C., De Mares Maryam C., van Elsas J.D. (2014). The mycosphere constitutes an arena for horizontal gene transfer with strong evolutionary implications for bacterial-fungal interactions. FEMS Microbiol Ecol.

[53] Mousa W.K., Shearer C., Limay-Rios V., Ettinger C.L., Eisen J.A., Raizada M.N. (2016). Root-hair endophyte stacking in finger millet creates a physicochemical barrier to trap the fungal pathogen Fusarium graminearum. Nat Microbiol.

[54] Fonteyne S., Castillo Caamal J.B., Lopez-Ridaura S., Van Loon J., Espidio Balbuena J., Osorio Alcalá L. (2023). Review of agronomic research on the milpa, the traditional polyculture system of Mesoamerica. Front Agron.

[55] Terán S., Rasmussen C.H. (1995). Genetic diversity and agricultural strategy in 16th century and present-day Yucatecan milpa agriculture. Biodivers Conserv.

[56] De La Torre S.J.F., González S.R., Cruz G.E.J., Pichardo G.J.M., Quintana C.M., Contreras T.A.R. (2018). Crop wild relatives in Mexico: an overview of richness, importance, and conservation status. North Am Crop Wild Relat Vol 1 Conserv Strateg.

[57] Gastélum G., Ángeles-Morales A., Arellano-Wattenbarger G., Guevara-Hernandez E., Rocha J. (2024). Biofilm formation and maize root-colonization of seed-endophytic Bacilli isolated from native maize landraces. Appl Soil Ecol.

[58] Gastélum G., Aguirre‐von‐Wobeser E., de la Torre M., Rocha J. (2022). Interaction networks reveal highly antagonistic endophytic bacteria in native maize seeds from traditional milpa agroecosystems. Environ Microbiol.

[59] Arellano-Wattenbarger G.L., Aguirre-Von Wobeser E., de la Torre M., Rocha J. (2023). Contribution of seed-endophytic bacteria to drought tolerance in early developmental stages of native maize landraces from arid milpas. Plant Soil.

[60] Flemming H.-C., Wingender J. (2010). The biofilm matrix. Nat Rev Microbiol.

[61] Collins G.S., Foias A.E. (2018). Maize goddesses and Aztec gender dynamics. Mater Cult Rev.

[62] Prjibelski A., Antipov D., Meleshko D., Lapidus A., Korobeynikov A. (2020). Using SPAdes de novo assembler. Curr Protoc Bioinforma.

[63] Chklovski A., Parks D.H., Woodcroft B.J., Tyson G.W. (2023). CheckM2: a rapid, scalable and accurate tool for assessing microbial genome quality using machine learning. Nat Methods.

[64] Gurevich A., Saveliev V., Vyahhi N., Tesler G. (2013). QUAST: quality assessment tool for genome assemblies. Bioinformatics.

[65] Rodriguez-R L.M., Gunturu S., Harvey W.T., Rosselló-Mora R., Tiedje J.M., Cole J.R. (2018). The Microbial Genomes Atlas (MiGA) webserver: taxonomic and gene diversity analysis of Archaea and Bacteria at the whole genome level. Nucleic Acids Res.

[66] Rodriguez‐R L.M., Harvey W.T., Rosselló‐Mora R., Tiedje J.M., Cole J.R., Konstantinidis K.T. (2015). Classifying prokaryotic genomes using the microbial genomes Atlas (MiGA) webserver. Bergeys Man Syst Archaea Bact.

[67] Meier-Kolthoff J.P., Göker M. (2019). TYGS is an automated high-throughput platform for state-of-the-art genome-based taxonomy. Nat Commun.

[68] Burkholder P.R., Pfister R.M., Leitz F.H. (1966). Production of a pyrrole antibiotic by a marine bacterium. Appl Microbiol.

[69] Merritt J.H., Kadouri D.E., O'Toole G.A. (2011). Growing and analyzing static biofilms. Curr Protoc Microbiol.

[70] Mielich‐Süss B., Lopez D. (2015). Molecular mechanisms involved in B acillus subtilis biofilm formation. Environ Microbiol.

[71] Ray V.A., Morris A.R., Visick K.L. (2012). A semi-quantitative approach to assess biofilm formation using wrinkled colony development. J Vis Exp.

[72] Arnaouteli S., Bamford N.C., Stanley-Wall N.R., Kovács Á.T. (2021). Bacillus subtilis biofilm formation and social interactions. Nat Rev Microbiol.

[73] Branda S.S., González-Pastor J.E., Ben-Yehuda S., Losick R., Kolter R. (2001). Fruiting body formation by Bacillus subtilis. Proc Natl Acad Sci.

[74] Ostrowski A., Mehert A., Prescott A., Kiley T.B., Stanley-Wall N.R. (2011). YuaB functions synergistically with the exopolysaccharide and TasA amyloid fibers to allow biofilm formation by Bacillus subtilis. J Bacteriol.

[75] Veening J.-W., Kuipers O.P., Brul S., Hellingwerf K.J., Kort R. (2006). Effects of phosphorelay perturbations on architecture, sporulation, and spore resistance in biofilms of Bacillus subtilis. J Bacteriol.

[76] Vlamakis H., Aguilar C., Losick R., Kolter R. (2008). Control of cell fate by the formation of an architecturally complex bacterial community. Genes Dev.

[77] Beauregard P.B., Chai Y., Vlamakis H., Losick R., Kolter R. (2013). Bacillus subtilis biofilm induction by plant polysaccharides. Proc Natl Acad Sci.

[78] Dragoš A., Kiesewalter H., Martin M., Hsu C.-Y., Hartmann R., Wechsler T. (2018). Division of labor during biofilm matrix production. Curr Biol.

[79] Kirisits M.J., Prost L., Starkey M., Parsek M.R. (2005). Characterization of colony morphology variants isolated from Pseudomonas aeruginosa biofilms. Appl Environ Microbiol.

[80] Yip E.S., Geszvain K., DeLoney‐Marino C.R., Visick K.L. (2006). The symbiosis regulator RscS controls the syp gene locus, biofilm formation and symbiotic aggregation by Vibrio fischeri. Mol Microbiol.

[81] Auch A.F., von Jan M., Klenk H.-P., Göker M. (2010). Digital DNA-DNA hybridization for microbial species delineation by means of genome-to-genome sequence comparison. Stand Genomic Sci.

[82] Richter M., Rosselló-Móra R. (2009). Shifting the genomic gold standard for the prokaryotic species definition. Proc Natl Acad Sci.

[83] Bochkareva O.O., Moroz E.V., Davydov I.I., Gelfand M.S. (2018). Genome rearrangements and selection in multi-chromosome bacteria Burkholderia spp. BMC Genom.

[84] Teng J.L., Yeung M.L., Chan E., Jia L., Lin C.H., Huang Y. (2017). PacBio but not Illumina technology can achieve fast, accurate and complete closure of the high GC, complex Burkholderia pseudomallei two-chromosome genome. Front Microbiol.

[85] Madsen J.S., Røder H.L., Russel J., Sørensen H., Burmølle M., Sørensen S.J. (2016). Coexistence facilitates interspecific biofilm formation in complex microbial communities. Environ Microbiol.

[86] Foster K.R., Bell T. (2012). Competition, not cooperation, dominates interactions among culturable microbial species. Curr Biol.

[87] McClean D., Friman V., Finn A., Salzberg L.I., Donohue I. (2019). Coping with multiple enemies: pairwise interactions do not predict evolutionary change in complex multitrophic communities. Oikos.

[88] Rivett D.W., Scheuerl T., Culbert C.T., Mombrikotb S.B., Johnstone E., Barraclough T.G. (2016). Resource-dependent attenuation of species interactions during bacterial succession. ISME J.

[89] Vlamakis H., Chai Y., Beauregard P., Losick R., Kolter R. (2013). Sticking together: building a biofilm the *Bacillus subtilis* way. Nat Rev Microbiol.

[90] Herren C.M., McMahon K.D. (2017). Cohesion: a method for quantifying the connectivity of microbial communities. ISME J.

[91] Pascual-García A., Bonhoeffer S., Bell T. (2020). Metabolically cohesive microbial consortia and ecosystem functioning. Philos Trans R Soc B.

[92] Blondel J. (2003). Guilds or functional groups: does it matter?.

[93] Lebeis S.L. (2015). Greater than the sum of their parts: characterizing plant microbiomes at the community-level. Curr Opin Plant Biol.

[94] Romdhane S., Spor A., Aubert J., Bru D., Breuil M.-C., Hallin S. (2022). Unraveling negative biotic interactions determining soil microbial community assembly and functioning. ISME J.

[95] Merritt J.H., Brothers K.M., Kuchma S.L., O'Toole G.A. (2007). SadC reciprocally influences biofilm formation and swarming motility via modulation of exopolysaccharide production and flagellar function.

[96] López D., Vlamakis H., Kolter R. (2010). Biofilms. Cold Spring Harbor Perspect Biol.

[97] Nadell C.D., Bucci V., Drescher K., Levin S.A., Bassler B.L., Xavier J.B. (2013). Cutting through the complexity of cell collectives. Proc R Soc B Biol Sci.

[98] Nadell C.D., Xavier J.B., Foster K.R. (2008). The sociobiology of biofilms. FEMS Microbiol Rev.

