# Supplementary material for: Harnessing emergent properties of microbial consortia for Agriculture: Assembly of the Xilonen SynCom
Source: Biofilm. 2025 May 3;9:100284. doi: 10.1016/j.bioflm.2025.100284 (PMC12127623; doi:10.1016/j.bioflm.2025.100284)
Supplement: Multimedia component 1 [file mmc1.pdf]

Supplementary material for:

Harnessing Emergent Properties of Microbial Consortia for Agriculture: Assembly of the Xilonen SynCom

Supplementary tables

Supplementary Table S1. Bacterial strains used for the combinatorial screening of interactions.

Code	Strain	Reference
NME9	*Bacillus* sp.	(1)
NME26	*Bacillus* sp.	
NME32	*Domibacillus* sp.	
NME36	*Bacillus* sp.	
NME37	*Domibacillus* sp.	
NME52	*Bacillus* sp.	
NME63	*Bacillus* sp.	
NME85	*Bacillus* sp.	
NME100	*Paenibacillus* sp.	
NME101	*Bacillus* sp.	
NME117	*Bacillus* sp.	
NME135	*Peribacillus* sp.	
NME155	*Bacillus* sp.	
NME186	*Paenibacillus* sp.	
NME233	*Bacillus* sp.	
NME235	*Paenibacillus* sp.	
NME239	*Paenibacillus* sp.	
NME246	*Bacillus* sp.	
NME247	*Peribacillus* sp.	
XM5	*Burkholderia* sp.	(2)
XM7	*Burkholderia* sp.	
XM13	*Burkholderia* sp.	
GW1	*Pseudomonas* sp.	(3)
GW6	*Pseudomonas* sp.	
GW9	*Pseudomonas* sp.	
GW12	*Pseudomonas* sp.	

Supplementary Table S2. Genomic sequencing, assembly, and annotation statistics of members of the *Xilonen* SynCom

	Species	***Bacillus pumilus* NME155**	***Bukholderia contaminans* XM7**	***Pseudomonas* sp. GW6**
**Before filtering**	**Total reads**	1,258,066	3,420,686	1,475,926
	**Mean reads length (bases)**	112	100 (fwd), 101 (rev)	119
	**Total bases**	142,052,912	345,156,917	176,310,205
	**Q20 bases (%)**	97.75	97.08	97.58
	**Q30 bases (%)**	96.99	96.14	96.75
**After filtering**	**Total reads**	1,246,602	3,342,712	1,456,018
	**Mean reads length (bases)**	93	85 (fwd), 84 (rev)	102
	**Coverage (×)***	31	56.04	27
	**Total bases**	116,973,366	282,717,490	149,161,164
	**Q20 bases (%)**	98.01	97.93	98.01
	**Q30 bases (%)**	97.31	97.08	97.24
**Assembly**	**Assembled genome size (bp)**	3,796,551	5,069,692	5,488,062
	**Number of contigs**	160	8,997	574
	**N50 value**	97,169	1,117	25,285
	**L50 value**	12	644	66
	**GC content (%)**	41.59	61.63	62.5
	**Completeness (%)**	100	66.39	100
	**Contamination (%)**	0.03	7.48	0.38
**Identification**	**Closest type strain (NCBI RefSeq assembly no.)**	GCF_900186955.1	GCF_000987075.1	GCF_002741105.1
	**Average Nucleotide Identity ANI (%)**	95.36	99.58	91.89
	**digital DNA-DNA Hybridization dDDH (%)**	63.9	98.9	44.8
**Annotation**	**Number of coding sequences**	4,056	**	5,489
	**Number of RNAs**	86	**	79
	**Annotated gene number**	1,385	**	1,591

Sequencing metrics shown here resulted after filtering and clean-up steps.
*, sequencing coverage was calculated as (total reads*mean read length)/assembled genome size
**, genome assembly was too low quality for annotation with RAST. Raw reads of NME155 and GW6 are deposited on the Sequence read Archive (SRA) of NCBI: https://www.ncbi.nlm.nih.gov/sra/PRJNA1197742

Supplementary figures

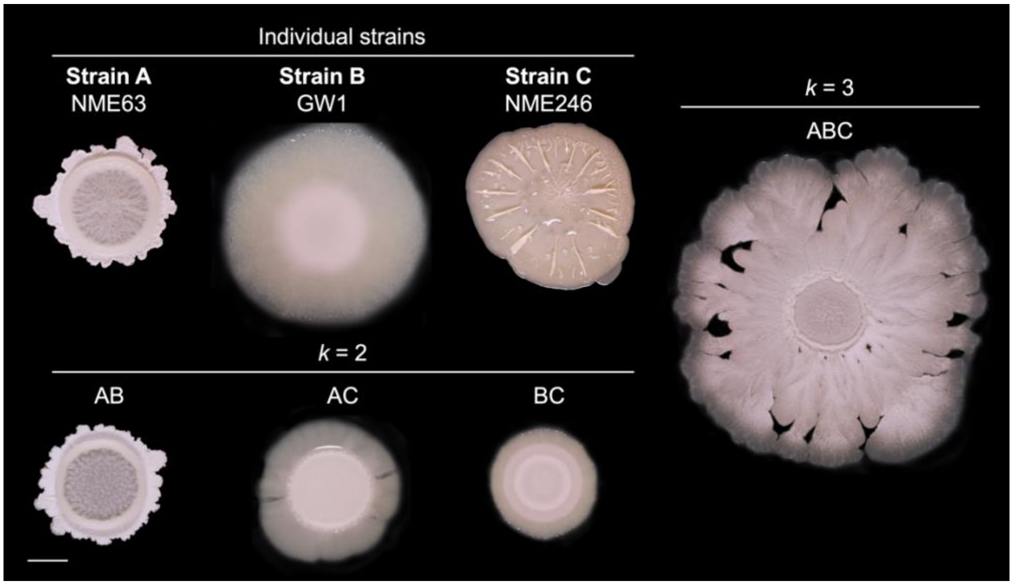

Figure S1. Community of *k* = 3 presenting emergent colony spreading. Colonies were grown in separate LB plates and pictures were taken after 3 days of incubation at 30 ºC. Scale bar: 5 mm.

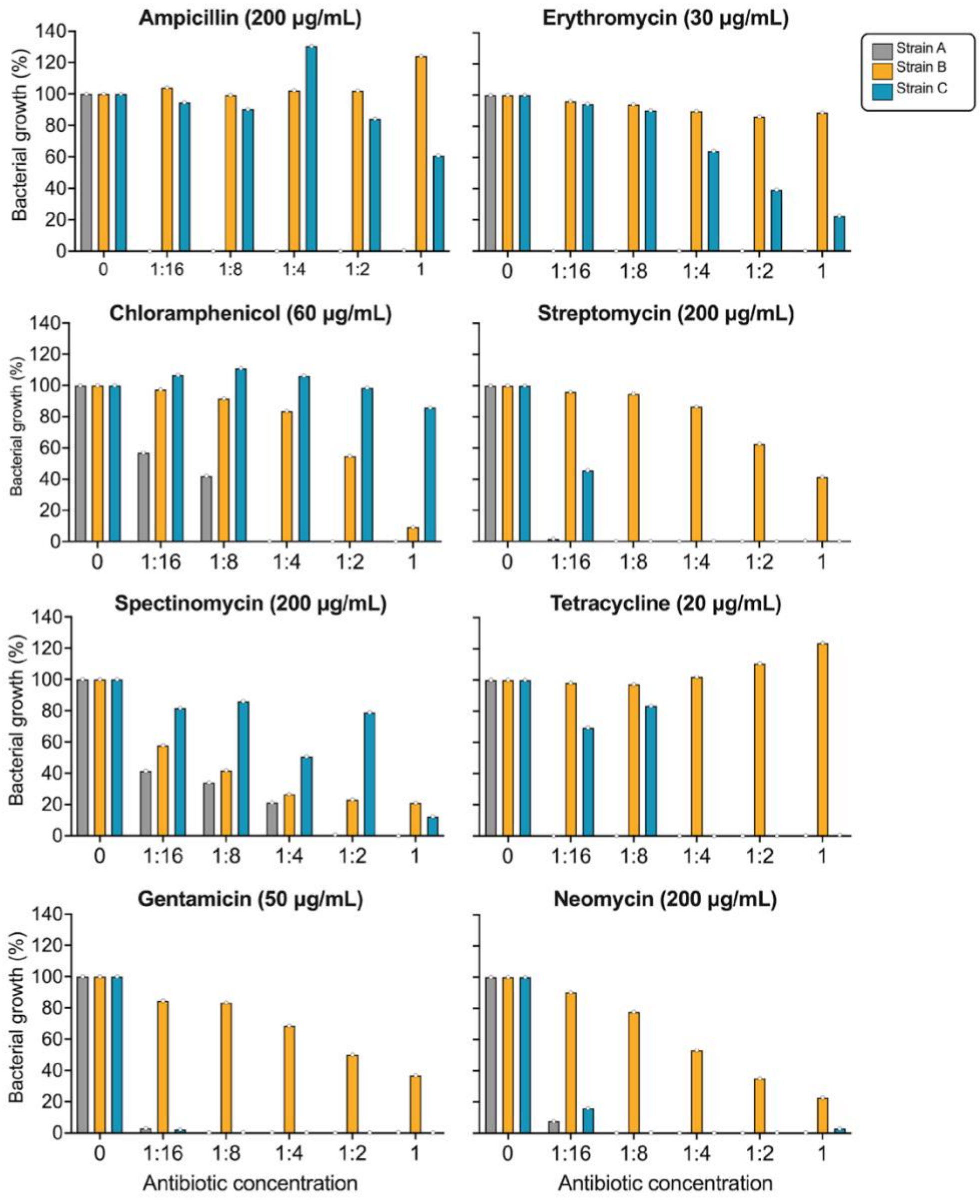

Supplementary Figure S2. Screening of antibiotic resistance for individual strains in the SynCom. Minimum inhibitory concentration assays were performed using 8 antibiotics and three strains. The highest concentration (identified as '1' on the X-axis) of each antibiotic is shown in parentheses. Strains were inoculated in 250 µl of fresh LB at a dilution factor of 1:500 from overnight liquid cultures previously washed with PBS. Microtiter plates were incubated without agitation at 25 ºC and Optical Density at 600 nm (OD_{600}) was measured after 3 days. Bacterial growth (OD_{600}) in antibiotics is shown as a percentage (%) of growth in LB media without antibiotics. Strain A: *Bacillus pumilus* NME155 (Bp_NME155); Strain B: *Burkholderia contaminans* XM7 (Bc_XM7) and Strain C: *Pseudomonas* sp. GW6 (P_GW6).

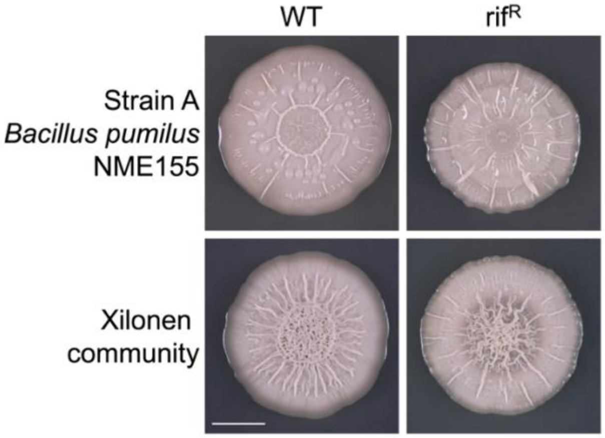

Supplementary Figure S3. Colony architecture of the *Xilonen* community using *Bacillus pumilus* NME155 Wild Type (WT) or the rifampicin-resistant variant (rif^R). Strains were grown in 20 mL of LB media at 30 ºC and 200 rpm for 16 h. Bacterial cultures were washed with PBS (see methods) and the optical density at 600 nm (OD_{600}) was adjusted to 1 ± 0.1. The community was assembled with either the WT or the rif^R variant of strain *B. pumilus* NME_155 by mixing the bacterial cultures at a 1:1:1 volume ratio, and 5 µL of each strain and the community were spotted on independent LB agar plates. Pictures were taken after 3 days of incubation at 30 ºC. Scale: 0.5 cm.

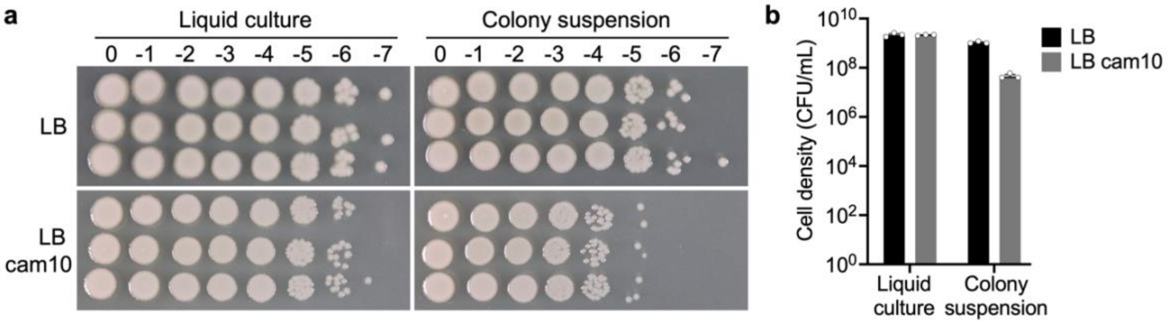

Supplementary Figure S4. Chloramphenicol resistance of *Pseudomonas* sp. GW6 (strain C) is decreased in colony biofilm, compared to liquid culture. a, CFU counts of *Pseudomonas* sp. GW6 were evaluated using 10-fold serial dilutions from triplicate liquid cultures and colony suspensions, which were spotted on LB and LB cam10. **b,** Cell density of liquid cultures and colony suspensions measured on LB and LB cam10. A washed overnight liquid culture was used for this experiment. For colony suspensions, a 3-day-old colony was used (5 µl spot inoculated in LB agar).

References

1. Gastélum G, Ángeles-Morales A, Arellano-Wattenbarger G, Guevara-Hernandez E, Rocha J. 2024. Biofilm formation and maize root-colonization of seed-endophytic Bacilli isolated from native maize landraces. Applied Soil Ecology 199:105390.
2. Gastélum G, Aguirre-von-Wobeser E, de la Torre M, Rocha J. 2022. Interaction networks reveal highly antagonistic endophytic bacteria in native maize seeds from

traditional milpa agroecosystems. Environmental Microbiology https://doi.org/10.1111/1462-2920.16189.
3. Arellano-Wattenbarger GL, Aguirre-Von Wobeser E, de la Torre M, Rocha J. 2023. Contribution of seed-endophytic bacteria to drought tolerance in early developmental stages of native maize landraces from arid milpas. Plant and soil 1–20.
